# Uncovering Novel QTLs and Candidate Genes for Salt Tolerance at the Bud Burst Stage in Rice through Genome-Wide Association Study

**DOI:** 10.3390/plants13020174

**Published:** 2024-01-08

**Authors:** Caijing Li, Changsheng Lu, Mengmeng Yang, Guangliang Wu, Mvuyeni Nyasulu, Haohua He, Xiaopeng He, Jianmin Bian

**Affiliations:** 1 Key Laboratory of Crop Physiology, Ecology and Genetic Breeding, Ministry of Education, Nanchang 330045, China; lcjkk20170409@163.com (C.L.); lcs71113@163.com (C.L.); mengyango2@163.com (M.Y.); glwu1996@163.com (G.W.); mvuyeni.nyasulu@gmail.com (M.N.); hhhua64@163.com (H.H.); 2 Institute of Agricultural Sciences, Ganzhou 341000, China

**Keywords:** rice, salt stress, bud burst stage, GWAS, candidate genes, haplotype

## Abstract

Salt stress is one of the most important factors limiting rice growth and yield increase. Salt tolerance of rice at the bud burst (STB) stage determines whether germinated seeds can grow normally under salt stress, which is very important for direct seeding. However, reports on quantitative trait loci (QTLs) and candidate genes for STB in rice are very limited. In this study, a natural population of 130 indica and 81 japonica rice accessions was used to identify STB-related QTLs and candidate genes using a genome-wide association study (GWAS). Nine QTLs, including five for relative shoot length (RSL), two for relative root length (RRL), and two for relative root number (RRN), were identified. Five of these STB-related QTLs are located at the same site as the characterized salt tolerance genes, such as *OsMDH1*, *OsSRFP1*, and *OsCDPK7*. However, an important QTL related to RSL, *qRSL1-2*, has not been previously identified and was detected on chromosome 1. The candidate region for *qRSL1-2* was identified by linkage disequilibrium analysis, 18 genes were found to have altered expression levels under salt stress through the RNA-seq database, and 10 of them were found to be highly expressed in the shoot. It was also found that, eight candidate genes (*LOC_Os01g62980*, *LOC_Os01g63190*, *LOC_Os01g63230*, *LOC_Os01g63280*, *LOC_Os01g63400*, *LOC_Os01g63460*, and *LOC_Os01g63580*) for *qRSL1-2* carry different haplotypes between indica and japonica rice, which exactly corresponds to the significant difference in RSL values between indica and japonica rice in this study. Most of the accessions with elite haplotypes were indica rice, which had higher RSL values. These genes with indica-japonica specific haplotypes were identified as candidate genes. Rice accessions with elite haplotypes could be used as important resources for direct seeding. This study also provides new insights into the genetic mechanism of STB.

## 1. Introduction

Rice (Oryza sativa L.) is one of the most important food crops, and feeds more than half of the world’s population [1]. Due to climate change, industrial pollution, and inadequate irrigation, the degree of soil salinity has become increasingly serious worldwide, and about 30% of the world’s rice-growing areas suffer from salt stress [2,3]. Salt stress has gradually become one of the major factors limiting rice growth and yield increase. Salt stress affects all stages of rice development and results in a low seed germination percentage and seedling growth rate, delayed seedling growth, wilting and browning of leaves, prolonged cropping duration, and reduced tillering [4,5,6]. When soil salinity increases, the water potential of the soil solution is lower than the water potential of plant root cells, resulting in an inhibition of water uptake by roots, and plants must make an osmotic adjustment to maintain cell expansion, growth, and water uptake. Osmotic stress also leads to stomatal closure, which inhibits carbon dioxide uptake and results in reduced photosynthesis. In addition, salt stress leads to the accumulation of reactive oxygen species in cells, which severely damage cell structures and macromolecules such as DNA, lipids, and enzymes.

Salt tolerance in rice is a typical quantitative trait controlled by the co-ordination of multiple genes and influenced by the external environment and developmental stage [7]. To date, more than 1,000 salt-tolerant QTLs and 140 genes for salt stress response have been identified on 12 chromosomes in all developmental stages [8,9]. At the germination and reproductive stages, more than 90 and 100 QTLs associated with salt stress have been identified using biparental populations and GWAS methods, respectively [10,11,12,13,14,15,16,17,18], and some genes such as *OsHsp17.0* [19], *OsbHLH035* [20], *qSE3* [21], and *OsNAC45* [22] have been characterized. In addition, most of the QTLs discovered and salt-tolerant genes cloned so far are from the seedling stage. For example, *SKC1* [23], *OsHAK21* [24], *HST1* [25], and *DST* [26] are the major genes associated with the seedling stage under salt stress. Although so many QTLs and genes have been identified and characterized, few studies have focused on salt tolerance in rice at the bud burst stage.

In recent years, the direct seeding cultivation of rice has gradually replaced traditional transplanting in many countries due to its advantages such as time and labor saving, and low cost [27]. The germinated seed is often planted directly in the fields, which imposes higher requirements on the smooth seedling formation of germinated seed under various abiotic stresses. However, few studies have mapped QTLs for STB. Four QTLs were detected during the bud burst stage in a chromosome segment substitutionline that was derived from a cross between the japonica rice accession Nipponbare and the indica rice accession 9311 [28]; five QTLs related to relative shoot length were detected in an F_2:3_ population derived from a cross between IR36 and Weiguo at the bud burst stage using a QTL-seq method [8]; and four QTLs were identified for STB using a high-density genetic map from a cross between Kongyu131 and Xiaobaijingzi [29]. Therefore, it is of great importance to explore as many QTLs and causal genes for STB as possible by using more germplasm resources.

Compared with the traditional QTL mapping method, which is not very efficient, GWAS can provide information on variation in complex traits, quickly identify the variation loci related to traits, and achieve great success in efficiently identifying the association of new variant traits. In this study, QTLs associated with STB were detected by GWAS using a natural population of 130 indica and 81 japonica rice accessions. A total of nine QTLs were identified, and their PVE values ranged from 6.08% to 9.24%. In a new major QTL, *qRSL1-2*, 18 differentially expressed genes were identified, of which some differentially expressed genes with indica-japonica-specific haplotypes were considered as candidate genes for STB. These results will be useful for improving STB and developing direct seeding of rice in saline soil.

## 2. Results

### 2.1. Phenotypic Variation in the 211 Rice Varieties

**Figure 1:**
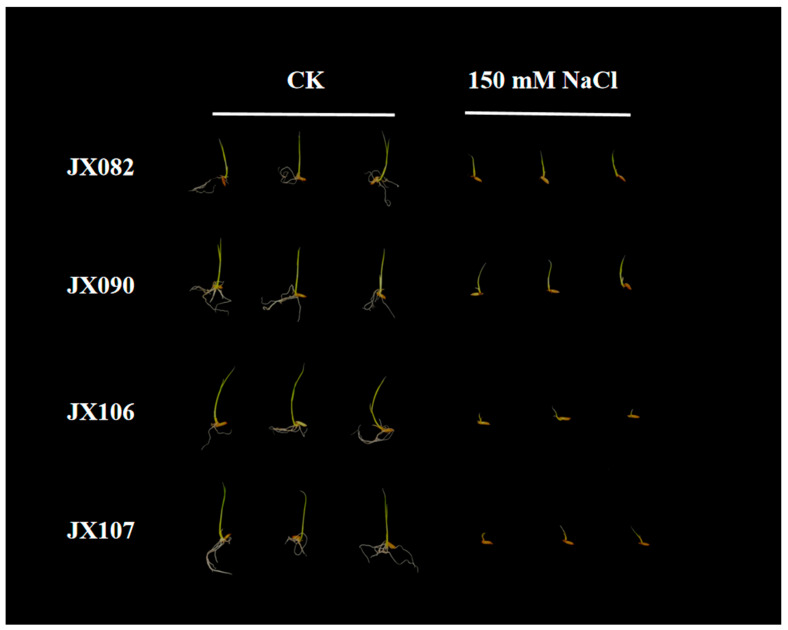
Phenotypic displays of four randomly selected rice varieties from the natural population after seven days of control and salt stress.

**Figure 2:**
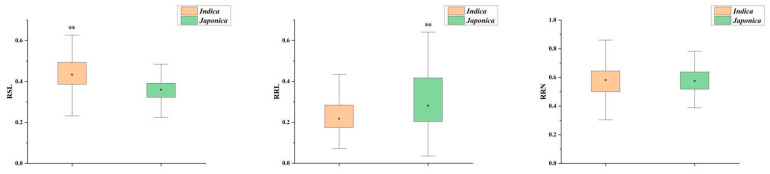
Comparison of STB indices among different subgroups. Yellow boxes represent indica, green boxes represent japonica, and black dots in the boxes represent average values. ** indicates significance at the 1% level.

In this study, a natural population of 130 *indica* and 81 *japonica* rice accessions was used to evaluate STB. Phenotypic displays of four randomly selected rice accessions showed that STB varied widely among 211 rice accessions after a seven-day salt stress treatment (Figure 1). The means, coefficients of variation, and ranges of RSL, RRL, and RRN at the bud burst stage of 211 rice accessions are presented in Table 1. The values of the three indices of the natural populations were all <1, indicating that the phenotypic traits of rice were inhibited by salt stress. The variation for RSL, RRL, and RRN among the 211 rice accessions ranged from 0.231 to 0.696, 0.035 to 0.910, and 0.318 to 0.86, respectively. The coefficient of variation (CV) of RRL and the mean value of RRN were the highest among the three indices. In addition, there were significant differences in RSL and RRL between the indica and japonica subgroups (Table 1, Figure 2). The RSL value of indica was significantly higher than that of japonica; in contrast, the RRL value of japonica was significantly higher than that of indica, and there was no significant difference in RRN value between the two subgroups. Correlation analyses among the three indices showed that RSL, RRL, and RRN were positively correlated with each other (Table 2). Compared with RRL, RSL and RRN are more likely to share the same genetic pathway because they are more highly correlated.

### 2.2. Identification of QTLs for STB via GWAS

**Figure 3:**
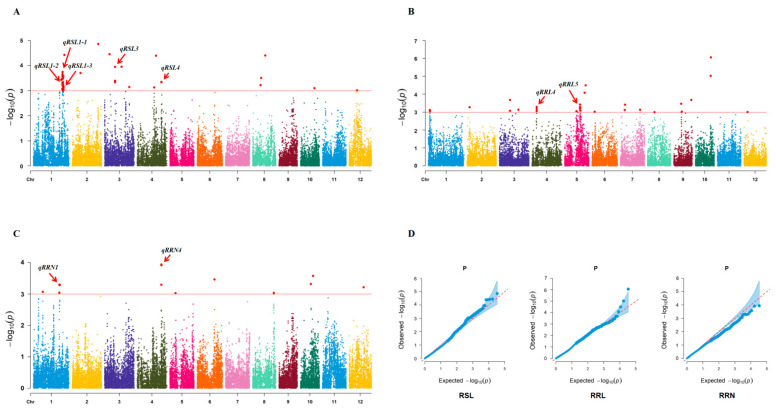
Manhattan plots and Q–Q plot (**D**) of GWAS for three STB indices. The red arrow indicates QTLs detected from RSL (**A**), RRL (**B**) and RRN (**C**). The solid red line in (**A**–**C**) is the threshold line. The red dashed line in (**D**) is the calibration line.

Based on the genotype (36,727 SNPs) and phenotype data (Table S1), GWAS was performed with a mixed linear model using PCA and KINSHIP results as covariates. A total of nine QTLs (*qRSL1-1*, *qRSL1-2*, *qRSL1-3*, *qRSL3*, *qRSL4*, *qRRL4*, *qRRL5*, *qRRN1*, and *qRRN4*) were identified in STB with well-fitted quantile-quantile (Q-Q) plots (*p* < 0.001) (Figure 3, Table 3). On chromosome 1, a QTL named *qRSL1-1* overlapped with *OsMDH1* [30] and *qRRN1* was colocalized with QTL_2 [31]. The other two QTLs (*qRSL1-2* and *qRSL1-3*) on chromosome 1 have not been previously reported. The QTL on chromosome 3, *qRSL3*, explains the greatest phenotypic variation and is co-localized with *OsSRFP1* [32]. Two QTLs on chromosome 4, *qRSL4* and *qRRN4*, were found to share the same peak SNP, and their candidate region contained a cloned salt stress-related gene, *OsCDPK7* [33]. Another QTL on chromosome 4, *qRRL4*, has not been previously reported. Similarly, a QTL at 18.8 Mb on chromosome 5, called *qRRL5*, has never been reported before. Among these QTLs, the phenotypic variance explained (R^2^) ranged from 6.78% for *qRRN1* to 9.24% for *qRSL3*.

### 2.3. Candidate Genes Screen in qRSL1-2

**Figure 4:**
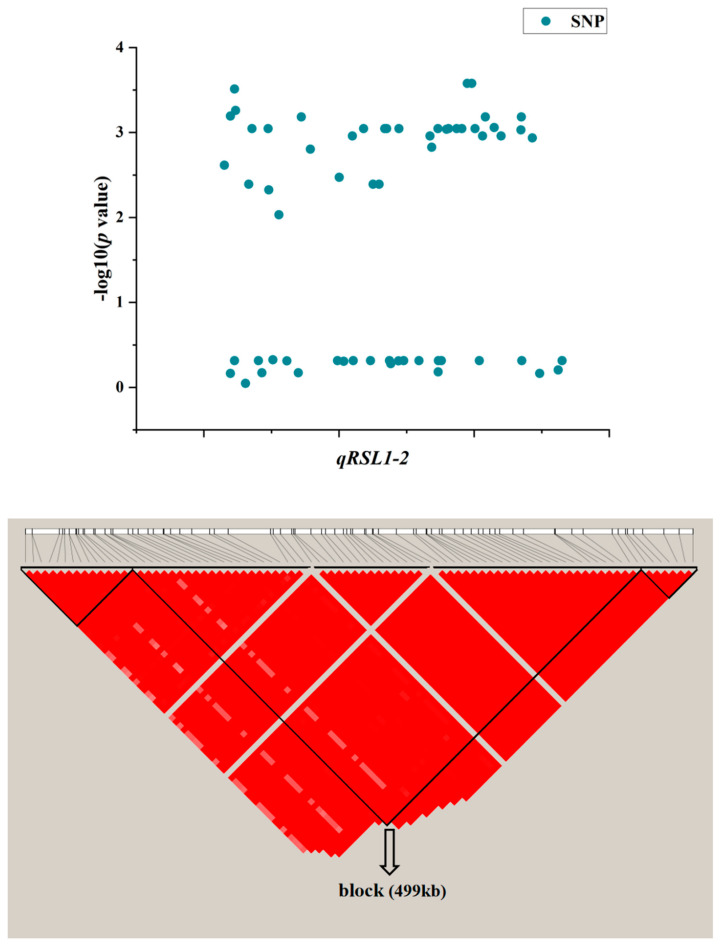
LD block of candidate region estimation of *qRSL1-2*.

To screen valuable candidate genes for salt tolerance, a QTL that explained the greatest phenotypic variation among the four newly found QTLs, *qRSL1-2*, was used to perform candidate gene analysis. After LD decay analysis, a total region of 499 kb was identified as a candidate region for *qRSL1-2* (Figure 4), and the candidate region contained 69 genes, including 31 functionally annotated genes, 11 expressed proteins of unknown function, 15 retrotransposon proteins, 10 transposon proteins and two hypothetical proteins (Table S2). To narrow down the number of candidate genes, genes categorized as expressed protein, hypothetical protein, retrotransposon, and transposon were discarded. Among the 31 functionally annotated genes, 18 genes were found to have significant differences in expression levels between control and salt treatments according to the Plant Public RNA-seq Database (|logFoldChange| ≥ 1.5), of which nine genes were up-regulated under salt stress, five genes were down-regulated, and the other four genes had different expression trends under the different projects (Table S3). In addition, the expression patterns of these 18 differentially expressed genes were identified using the Rice Genome Annotation Project and the Plant Public RNA-seq database, and 10 differentially expressed genes were found highly expressed in shoots (Table 4), suggesting that these 10 genes could be candidates for *qRSL1-2*.

### 2.4. Natural Allelic Variation in Candidate Genes Contributes to RSL

**Figure 5:**
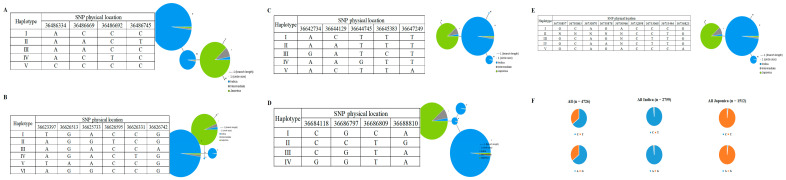
Haplotype analysis of seven genes (*LOC_Os01g62980*/ (**A**), *LOC_Os01g63190/* (**B**), *LOC_Os01g63230/* (**C**), *LOC_Os01g63280/* (**D**), *LOC_Os01g63400/* (**E**), *LOC_Os01g63460,* and *LOC_Os01g63580/* (**F**)) with non-synonymous SNPs in the coding region.

As mentioned earlier, indica rice had higher RSL than japonica rice. Therefore, we investigated whether variations in the alleles of candidate genes contributed to this difference. According to RiceVarMap v2.0, the sequences of these 10 genes from *qRSL1-2* were compared in 4726 rice varieties. The variable sites in the promoter region or the non-synonymous SNPs from the coding region of seven of the 10 genes had *indica*-*japonica* specificity. One LTP family protein, *LOC_Os01g62980*, had four non-synonymous SNPs, vg0136486334 (A/C), vg0136486669 (A/C), vg0136486692 (C/T), and vg0136486745 (C/T) in the coding region. Five different haplotypes were identified, including the three major haplotypes, Hap I, Hap II, and Hap III. Most indica rice has haplotypes II and III, while japonica rice has haplotype I (Figure 5A). The gene *LOC_Os01g63190*, had a total of six nonsynonymous SNPs, namely vg0136623397 (A/T), vg0136625733 (G/A), vg0136626331 (T/C), vg0136626513 (G/A), vg0136626595 (C/T), and vg0136626742 (G/A). Six different haplotypes, including three major haplotypes, Hap I, Hap II, and Hap IV, were identified based on the six non-synonymous SNPs in cultivated rice and exhibited a large genetic difference between indica and japonica (Figure 5B). There are five non-synonymous SNPs in *LOC_Os01g63230*, vg0136642734 (A/G), vg0136644129 (A/C), vg0136644745 (T/G), vg0136645383 (T/C), and vg0136647249 (T/A). Five major haplotypes were observed at *LOC_Os01g63230*, which encodes a growth regulatory protein. Hap I and V occurred in japonica, whereas Hap II, III, and IV occurred mainly in indica (Figure 5C). A total of four non-synonymous SNPs (vg0136684118, vg0136686797, vg0136686809, and vg0136688810) in *LOC_Os01g63280* identified four haplotypes (Figure 5D). The vast majority of japonica have Hap I, and indica rice has Hap II, Hap III, and Hap IV. There are nine non-synonymous SNPs in the coding region of the gene *LOC_Os01g63400*, which make up five haplotypes. Indica rice contains mainly Hap II and III, whereas japonica rice contains mainly Hap I (Figure 5E). Interestingly, we found that the coding regions of *LOC_Os01g63460* and *LOC_Os01g63580* have only one non-synonymous SNP, vg0136765004 (C/T) and vg0136864077 (A/G), respectively. Indica rice contains mainly C and A, while japonica rice contains mainly Hap T and G (Figure 5F). Taken together, these results suggest that the natural allelic variation in these eight candidate genes appears to be related to the different RSLs observed among the 211 rice accessions.

## 3. Discussion

To our knowledge, this is the first report of excavation of STB-related QTLs and candidate genes using GWAS. The first problem we need to solve is which indicators can be efficiently used to assess STB in the natural population. At the bud burst stage, the survival rate of rice seeds under various abiotic stresses and the growth status of the rice seed bud and root system are important indices for evaluating the abiotic stress tolerance of rice. In recent years, studies on abiotic stress tolerance at the bud burst stage mainly focused on cold and salt, and almost all studies used seedling survival rate to evaluate cold tolerance at the bud burst stage [34,35,36,37,38,39,40]. At the same time, RSL, RRL, RRN and seedling survival rate were used to evaluate the salt and alkali tolerance of rice at the bud burst stage [8,28,29,41]. Significant differences in the use of RSL, RRL, and RRN to assess STB were observed in the study, with maximum RSL and RRN index values approximately three times higher than minimum RSL and RRN index values, and the maximum RRL values as high as twenty-six times the minimum RRL values (Table 1). Comparing the three index differences and correlations between the two subgroups in the population, it was found that the RSL value of indica rice was significantly higher than that of japonica rice, while the RRL value of japonica rice was significantly higher than that of indica rice and the RSL value of the population was significantly positively correlated with RRL. These differences might be due to the different genetic mechanisms of indica and japonica rice in responding to salt stress. Our phenotypic identification results may provide new insights into the genetic differentiation of salt stress responses in indica/japonica subgroups during evolution.

Traditional QTL mapping methods are limited by the genetic diversity of the parents, often fail to identify variant loci, and often take a long time to establish a set of biparental populations. Due to the availability of high-density SNP maps and diverse rice germplasm resources, GWAS has become a mature and efficient strategy to identify the loci of variation in related traits. In the current study, nine STB-related QTLs were discovered via GWAS. Among these QTLs, five QTLs have been previously reported. A cloned salt stress-related gene, *OsMDH1* (*LOC_Os01g61380*), is located in the candidate region of *qRSL1-1*. *OsMDH1* encodes a malate dehydrogenase expressed in various tissues, including the bud, and the loss-of-function mutant shows a salt tolerant phenotype [30]. The QTL *qRSL3* overlapped with *OsSRFP1*, a gene encoding an E3 ubiquitin ligase localized in the cytoplasm and nucleus; *OsSRFP1* is expressed in several organs of rice and is highly expressed in roots, nodules and panicles and is also induced by low temperature, dehydration, salt, H_2_O_2_, and abscisic acid [32]. A calcium-dependent protein kinase, *OsCDPK7*, appeared in a QTL region repeatedly detected in our study (i.e., *qRSL4*/*qRRN4*), and the tolerance of *OsCDPK7*-overexpressedplants to low temperature, high salinity, and drought stress was significantly increased [33]. The same QTL was located in the two different indices of RSL and RRN, which accurately supports the suggestion mentioned earlier that RSL and RRN may share common genetic pathways. One RRN-related QTL, *qRRN1*, was localized in our study along with *QTL_2*, a previously reported QTL for salt stress mediating chlorophyll content [31]. In addition, four QTLs were reported for the first time in our study. Whether the candidate regions of these QTLs contain causal genes for STB is worth further discussion.

One QTL related to RSL, *qRSL1-2*, was selected for further screening of candidate genes. By analyzing linkage disequilibrium, screening differentially expressed genes, and analyzing expression patterns, 10 genes were found to be highly expressed in the shoot response to salt stress. As mentioned earlier, RSL values differed significantly between indica and japonica subgroups, suggesting that there may be sequence differences between indica and japonica subgroups in candidate genes that determine the shoot growth of germinated seeds under salt stress. Therefore, SNPs that may alter protein function were sought from the coding regions and promoters of these ten genes, and the results showed that haplotypes of seven genes exhibited indica-japonica specificity. Among these seven genes, *LOC_Os01g62980* encoding non-specific lipid transfer protein d5 and was found to be involved in drought stress and stimulated by some exogenous hormones [42,43]. In secondary metabolism, a laccase gene, *LOC_Os01g63190*, was found to be an HT salt-responsive gene [44]. The receptor-like cytoplasmic kinase (*OsRLCK*) gene *LOC_Os01g63280* (*OsRLCK48*) has been reported to be down-regulated under salt stress [45]. In conclusion, these differentially-expressed genes with indica-japonica specific haplotypes isolated from *qRSL1-2* can be used as candidate genes for STB, which will be further verified in subsequent experiments.

## 4. Materials and Methods

### 4.1. Plant Materials

The natural population used in this study consisted of 130 indica and 81 japonica accessions. These accessions were mainly collected from 15 different provinces in China, as well as the Philippines and Japan. Their geographical range extends from 15° to 48° north in latitude, including temperate, tropical, and subtropical regions [40]. The detailed information for each rice accession in the population, including number, name, origin, and subgroup type, is shown in Table S1. The rice material was collected in accordance with local laws and without conflicts of interest. The population was developed in the experimental field of Jiangxi Agricultural University in Nanchang, Jiangxi Province, and Linwang, Hainan Province, for more than four generations.

### 4.2. Salt Tolerance Evaluation at the Bud Burst Stage

Mature seeds harvested from the field were placed in a 45 °C oven for 2 days to break seed dormancy. The seeds with broken dormancy were disinfected with sodium hypochlorite and 75% ethanol, and then washed three times with sterile water for the experiment. The 30 plump seeds were germinated in 9 cm diameter Petri dishes with 15 mL sterile water until the buds had a length of about 5 mm in length. All seeds with a bud length of 5 mm were grown in a new Petri dish to which 15 mL of 150 mM NaCl had been added. At this time, control experiments were performed simultaneously. Seven days later, 10 seedlings were selected in each dish and shoot length, root length and number of roots were measured. Relative value = value after salt treatment/value of the control group. The salt solution was replaced every day. The experiment was repeated three times.

### 4.3. Genome-Wide Association Study

Genome-wide association study (GWAS) analysis was performed on SNPs and phenotypes using Tassel 5.2.73 software, and the mixed linear model (MLM) with the PCA matrix (the first five PCs were formed) and kinship (K matrix) were used to determine the association between SNP markers and the three phenotypic traits. To ensure data were accuracy, the phenotypic data was standardized and SNP data (36,727 SNPs) were filtered before association mapping. Only SNPs that did not contain missing values and had a minor allele frequency (MAF) > 0.05 were used in the genotype data set. GWAS results were visualized with Q-Q plot and Manhattan plot using the R 4.1.0 package (CMplot). The P-value threshold was set at –log10(P) ≥ 3.0. Haplotype analysis was performed for candidate genes based on SNPs localized within coding regions.

### 4.4. Candidate Gene Analyses

LD blocks were run to identify candidate gene regions using Haploview 4.2 software, and the SNP with the most significant association in a block was identified as the leading SNP. LD blocks containing significantly associated SNPs were defined as a candidate genomic region. Differentially expressed genes were identified using the Plant Public RNA-seq Database [46]. Expression patterns of differentially expressed genes were analyzed using the Rice Genome Annotation Project (http://rice.uga.edu/index.shtml (accessed on 1 January 2022)). Haplotype analyses were performed using RiceVarMap V2.0 [47]. The information on candidate genes was collected and classified using NCBI (https://www.ncbi.nlm.nih.gov/ (accessed on 3 January 2022)) and China Rice Data Center (https://www.ricedata.cn/ (accessed on 3 January 2022)).

### 4.5. Genomic DNA Extraction, Sequencing and Genotyping

DNA was extracted from approximately 100 mg of fresh young leaves using the CTAB method, and the quantity and quality of DNA were measured using a Denovix DS-11 spectrophotometer (Denor, Atlanta, GA, USA). In addition, purity was determined via electrophoresis of DNA for 1 h at 60 V in a 1% agarose gel. The 50K rice gene SNP microarray ‘OsSNPNKs’ was used for genotyping. SNPs on the microarrays were evenly distributed across the genome, with an average distance of <1 kb between SNPs. Genotyping was based on Affymetrix AXIOM 2.0 (Thermo Fisher, Waltham, MA, USA) and the target prep protocol QRC (P/N 702990) kit (Thermo Fisher, Waltham, MA, USA) was used for DNA amplification, DNA fragmentation, microarray hybridization, DNA-binding single-base extension, and signal amplification. Staining and scanning were performed using a Gene Titan multichannel instrument (Thermo Fisher, Waltham, MA, USA).

### 4.6. Statistical Analysis

All the mean values and standard errors of phenotypic data were calculated using EXCEL 2010, and the correlation coefficients were calculated using SPSS 26.0 software. Box plots were produced using OriginPro 2021 software.

## 5. Conclusions

In this study, a natural population was measured using the three indices RSL, RRL, and RRN to evaluate STB. Based on this phenotypic data and 36,727 SNPs, a GWAS was performed. Based on the GWAS, a total of nine major QTLs were filtered out. In addition, haplotype difference analysis was performed for the differentially expressed genes in *qRSL1-2*. Finally, seven genes (*LOC_Os01g62980*, *LOC_Os01g63190*, *LOC_Os01g63230*, *LOC_Os01g63280*, *LOC_Os01g63400*, *LOC_Os01g63460*, and *LOC_Os01g63580*) were identified as candidate genes. The results of this study will enrich the gene pool of STB, deepen the understanding of the regulatory mechanism of STB, and provide a valuable reference for future molecular breeding work.

## Figures and Tables

**Table 1:** Phenotypic variation in three STB indices in *indica* and *japonica*.

Trait	211 Accessions			130 *indica* Accessions			81 *japonica* Accessions		
	Mean	Range	CV	Mean	Range	CV	Mean	Range	CV
RSL	0.415 ± 0.078	0.231–0.696	18.7%	0.443 ± 0.078	0.231–0.696	17.8%	0.371 ± 0.051	0.288–0.578	13.9%
RRL	0.276 ± 0.141	0.035–0.910	51.0%	0.251 ± 0.131	0.071–0.775	52.1%	0.316 ± 0.147	0.035–0.910	46.5%
RRN	0.584 ± 0.097	0.318–0.860	16.6%	0.580 ± 0.107	0.318–0.860	18.4%	0.591 ± 0.080	0.388–0.782	13.5%

**Table 2:** Correlation coefficients among STB indices.

Trait	RSL	RRL	RRN
RSL	1	0.267 **	0.432 **
RRL	0.267 **	1	0.204 **
RRN	0.432 **	0.204 **	1

**: Significant at *p* = 0.01.

**Table 3:** Summary of the significant SNPs detected via GWAS and the overlapping QTLs/genes reported previously.

QTL ID	Trait	Chr.	Peak SNPs	*p* Value	R^2^	Previous QTL/Genes
*qRSL1-1*	RSL	1	35691376	3.20 × 10^−4^	0.091007605	*OsMDH1*
*qRSL1-2*	RSL	1	36789943	2.65 × 10^−4^	0.082702247	
*qRSL1-3*	RSL	1	36988603	7.26 × 10^−4^	0.072572863	
*qRSL3*	RSL	3	13014108	1.13 × 10^−4^	0.092421651	*OsSRFP1*
*qRSL4*	RSL	4	29820476	4.53 × 10^−4^	0.077107238	*OsCDPK7*
*qRRL4*	RRL	4	5864214	5.18 × 10^−4^	0.076378954	
*qRRL5*	RRL	5	18874396	6.00 × 10^−4^	0.074780842	
*qRRN1*	RRN	1	32234318	5.06 × 10^−4^	0.060777188	*QTL_2*
*qRRN4*	RRN	4	29820476	1.15 × 10^−4^	0.092366087	*OsCDPK7*

**Table 4:** The RNA-Seq FPKM Expression Values of 10 DEGs.

Gene ID	Leaves-20 Days	Post-Emergence Inflorescence	Pre-Emergence Inflorescence	Anther	Pistil	Seed-5 DAP	Embryo-25 DAP	Endosperm-25 DAP	Seed-10 DAP	Endosperm- 25 DAP (Replicate)	Leaves-20 days (Replicate)	Shoots
*LOC_Os01g62950*	2.25939	10.0462	5.71393	216.92	8.45013	9.64699	2.03138	4.22441	3.58312	3.50467	3.7757	25.0827
*LOC_Os01g62980*	8.97772	55.7145	17.5485	1.64909	39.8088	5.31876	1.02588	0.550286	0.329246	0.332092	11.3404	45.3305
*LOC_Os01g63060*	7.51192	17.6248	11.2939	16.5555	40.2667	16.0646	8.30178	2.95839	4.79123	3.31343	7.28362	94.1526
*LOC_Os01g63190*	58.0209	39.3723	0.776449	0.709715	0	10.945	0	0	0.444664	0	11.7839	11.9054
*LOC_Os01g63230*	13.7457	6.46319	1.00233	5.34358	1.40058	0.672047	1.80477	13.6685	5.3863	13.6636	11.384	12.8333
*LOC_Os01g63280*	5.23959	1.04796	0.869788	1.10318	1.08601	0.832929	0	0	0	0	4.495	7.77339
*LOC_Os01g63400*	4.35858	5.53168	7.76593	0.69816	4.32159	1.941	3.98117	0.544953	0.73063	0.598556	1.50912	6.61778
*LOC_Os01g63460*	5.14934	7.66854	15.1861	1.18708	7.16601	4.26658	1.06111	0.32016	0	0	2.64417	6.98541
*LOC_Os01g63540*	0	0	3.28173	0	0	0	0	0	0	0	0	2.85494
*LOC_Os01g63580*	0.42975	78.9638	117.25	11.0498	44.6477	77.7596	6.86909	3.76347	3.26988	3.11506	1.19418	25.4433

## Data Availability

The data presented in this study are available within the article.

## Supplementary Materials

The following supporting information can be downloaded at https://www.mdpi.com/article/10.3390/plants13020174/s1, Table S1: Phenotypic data and information of the 211 rice varieties; Table S2: The genes in candidate region for qRSL1-2; Table S3: The differentially expressed genes for qRSL1-2 from RNA-seq database.
